# Maternal factors contributing to low birth weight deliveries in Tshwane District, South Africa

**DOI:** 10.1371/journal.pone.0213058

**Published:** 2019-03-01

**Authors:** Lumbani Tshotetsi, Loveness Dzikiti, Precious Hajison, Shingairai Feresu

**Affiliations:** 1 University of Pretoria, Faculty of Health Sciences, School of Health Systems and Public Health, Pretoria, South Africa; 2 University of Pretoria, Faculty of Health Sciences, Department of Family Medicine, Pretoria, South Africa; 3 PreLuHa consult, Zomba, Malawi; 4 University of Fort Hare, Faculty of Health Sciences, East London, South Africa; University of the Witwatersrand, SOUTH AFRICA

## Abstract

**Background:**

Low birth weight continues to be a main cause of child morbidity and mortality. Low birth weight can cause complications in adult life, and is therefore a public health concern. In this study, we determined the maternal factors that contribute to low birth weight (LBW) deliveries in Tshwane District, South Africa.

**Methods:**

We conducted a case control study of 1073 randomly selected mothers who delivered babies in four hospitals in the district. We reviewed antenatal and maternity registers to obtain information about the mothers and their offspring. We fitted a multiple logistic regression to examine relationships between possible factors associated with LBW.

**Results:**

From the total sample of mothers (n = 1073), 77% (n = 824) were adult women, aged 20 to 35 years. Of the adult mothers, 38.54% (n = 412) delivered low birth weight (LBW) infants. The mean gestational age and weight of all infants at birth was 37.16 weeks (SD 2.92) and 2675.48 grams (SD 616.16) respectively. LBW was associated with prematurity, odds ratio (OR) 7.15, 95% confidence interval (CI) 5.18 to 9.89; premature rupture of membranes OR 7.33, 95% CI 2.43 to 22.12 and attending fewer than five antenatal care (ANC) visits OR 1.30, 95% CI 1.06 to 1.61. Male infants were less likely to be LBW, in this population.

**Conclusion:**

Women who attended fewer than five ANC visits were predisposed to give birth to low birth weight babies. Mothers should be encouraged to attend ANC visits to detect adverse events like premature rupture of membranes and premature labour timeously.

## Introduction

Low birth weight (LBW–a baby born with less than 2500 grams) is a major cause of child morbidity and mortality, especially in sub-Saharan Africa, where most LBW babies are born [1]. Although data from Africa is not readily available, we know that treating LBW neonates is associated with high hospital expenditure in many countries [2]. The incidence of LBW deliveries has been associated with diverse factors [1], of which many are linked to a disadvantaged socio-economic status [3]. Low birth weight is an important public health concern than cannot be overlooked.

Babies can be born preterm or premature (<37 weeks) or they can be small for their gestational age (≥37 weeks but weigh < 2500 grams). Babies who are born preterm are prone to neonatal problems such as infection, which may require longer hospital stay, increased cost to the family and hospital, the government, and increased mortality rate [1]. Cost analyses studies on LBW are rare, though a study in Mozambique reported that the cost of raising a LBW baby was estimated at US$ 24.12 due to hospitalisation and caring for the baby; while the health system incurred US$ 169 957 [2].

The main reason for LBW is preterm delivery [1], but the aetiology of preterm delivery remains unknown. Most authors agree that preterm delivery can be caused by medical conditions, and infections like hypertension, malaria, syphilis, and HIV infection [4, 5]. A systematic review focussing on developing countries, found a strong association between maternal HIV infection and LBW [6]. Contrastingly, a randomised control trial (RCT) study in Malawi, failed to find an association between HIV and LBW, even though the prevalence of HIV was 26.2% [5]. Therefore, there is conflicting evidence on the relationship between HIV infection and LBW. Broek *et al* (2014) found that maternal anaemia was another risk factor for LBW (72.6% versus 64.5% in term pregnancy) [5]. A multinational RCT conducted in sub-Saharan Africa did not find any association between maternal anaemia and LBW, but maternal age of younger than 19 years and being malnourished were associated with LBW [7]. Kuar *et al* [8] compared adverse birth outcomes between anaemic and non-anaemic mothers, and found that maternal haemoglobin status was an importance predictor of neonate weight and length. One might argue that malnutrition, rather than just anaemia, in low-income countries, may affect the weight of neonates. Maternal nutrition affects the growth of the baby in utero and the eventual birth weight [9, 10]. Maternal infection may also limit the growth of the baby [11].

In sub-Saharan Africa, maternal malaria infection is an important predictor of adverse birth outcomes, including LBW or premature birth. In Congo, 94.5% of LBW babies were born to mothers who had malaria [12]. In Malawi, which is a malaria endemic area; a RCT showed that 36.4% women who had malaria delivered premature babies, compared to 28.5% women who did not have malaria [5]. Other causes that have been reported by different authors are non-communicable diseases such as hypertension or diabetes [13].

Maternal age is another unsettled issue, as it is unclear if young or adolescent mothers are more likely to give birth to LBW babies [13–15]. Some studies have suggested that young mothers (13 to 19 years) are more likely to deliver LBW infants, compared to adult mothers (20 to 45 years) [6, 14]. Ngoma *et al* (2016) found that teenage mothers had a higher risk of delivering a LBW infant than adult mothers (16% versus 9%) [7]. In contrast, Hoque and Hoque [16] compared the incidence of adverse obstetric and perinatal outcomes of adult mothers to teenage mothers. They found that slightly more teenage mothers (14.3%) than adult mothers (13.7%) gave birth to LBW babies (P = 0.56). Further binary logistic regression showed that teenage pregnancy did not predict a LBW outcome [17]. Similarly, in a Pretoria Tertiary Hospital, teenage mothers had 17.2% LBW babies compared to 12.6% of adult mothers (P = 0.140) and age did not predict LBW [16].

Globally, the prevalence of LBW is increasing and remains a challenge despite many interventions aimed at addressing this problem. The United Nations Children’s Fund (UNICEF) reported that in 2013, 22 million LBW infants were born globally, and most died in the neonatal period [18]. The World Health Organization (WHO) pointed out that most of LBW babies are born in low to middle-income countries, with 90% coming from sub-Saharan Africa [1]. UNICEF further reported, that most of LBW infants are born in informal delivery settings, where they are not weighed, thus making it difficult to get a true estimate of the magnitude of the problem [18]. We thus set out to describe the factors associated with LBW babies born to mothers in the Tshwane District of South Africa being one of the low to middle - income areas in Africa.

## Materials and methods

### Study setting

We selected our sample of mothers from four randomly selected hospitals in Tshwane District, South Africa, in 2014. In Gauteng province, Tshwane is one of the 5 districts that are monitored by the health systems trust (District health barometer) [19]. We used simple random selection to pick Tshwane district as our study area. The Tshwane District has a population of 3 243 597 and a population density of 515/km ^2^. It has seven regions, which have 68 clinics, 8 community health centres (CHC), 4 district hospitals, 1 regional hospital, 3 central hospitals and 27 other hospitals [19]. To select the study hospitals, we identified public community health centres and hospitals with maternity units in each region, and from these, randomly selected four facilities, namely Mamelodi Hospital, Pretoria West Hospital, Kgabo CHC and Laudium CHC.

### Data collection

We extracted and analysed a total data sample of 1073 mothers and their babies, with a 1:1 ratio of cases and controls. Cases were LBW infants; while controls were normal birth weight infants in this study.

All mothers and their babies who had delivered and or were referred to the healthcare centres between January 2013 and December 2014 were eligible for inclusion. We excluded mothers younger than 13 years and older than 35 years; mothers who had delivered more than 5 times (grand multipara); and mothers with twin babies since they may have affected the outcome of interest [11].

We determined the gestational age of the baby at birth from clinical records documented by the midwife. These records are usually obtained through self-reports of the mother’s last menstrual period, which the midwife and obstetrician use to estimate clinical gestational age. In South Africa, being a developing country, ultrasound is not readily available in most public health institutions.

We entered data, including maternal biographic, reproductive, medical and obstetrical information including the baby’s birth outcome using EpiData Software (http://www.epidata.dk).

We extracted all the data from antenatal files. Where information was not available, especially for women who were referred to another healthcare facility along with their antenatal files, we abstracted the information from maternity registers. We analysed the data using STATA 14 software. We computed descriptive statistics, that is, the frequencies of outcomes of interest. We used chi-square tests to identify significant associations between the outcome, LBW, and risk factors. We used a multiple logistic regression to model the odds of LBW by risk factors to determine whether associations existed. Continuous variables were analysed using t- test and presented as mean, standard deviation (SD) and p – values. A p-value less than 0.05, was considered statistically significant.

The variables that were used were birth weight (independent, coded 1 = LBW, 0 = NBW) and dependent variables were hypertension–clinically recorded by nurse or doctor in the case file during antenatal visit; preeclampsia–current hypertension with proteinuria (protein in urine) as clinically recorded; PROM–(already described above) this included all forms–preterm rupture of membranes before 37 weeks and prolonged rupture of membranes (PrlROM) before 37 weeks (did not include PrlROM after term gestation). Infection–urinary tract infection and uncategorised type as recorded in the case files. Anaemia–current low haemoglobin and or as stated in clinical records. Marital status–married–couples who were legally married, single–not legally married and not living with partner, stable relationship–living with partner in a stable relationship but not legally married. Residence–rural–living in squatter camps and or without basic amenities like water and electricity, semi-urban–living in location with basic amenities but in overcrowded locations, urban–living in suburb or within town. All these variables were derived from the clinical records as documented by health care workers.

### Ethical clearance

The study was approved by the University of Pretoria ethics committee (Ethics reference number 214/2016) and Department of Health Research Committee for Gauteng Province, South Africa (Project number 28/2016) prior to the study commencement. Facility managers of the four study sites gave written consent for the review of records. Confidentiality was ensured by anonymous data entry, and followed the Helsinki declaration for data management of retrospective study.

## Results

We included 1073 women who delivered at various study sites in Tshwane District. The mean age of the women was 24.18 (SD 5.13) years, 77% of whom were 20 years and older. The mean gestational age at first antenatal visit was 22.78 weeks (see Table 1), while the mean gestational age at birth was 37.16 weeks. The mean birthweight was 2675.48 grams, mean length of the infant was 48.86 centimetres, and mean head circumference was 33.21 cm. See Table 1 below

**Table 1:** Description of 1073 mothers who delivered at four Tshwane hospitals in 2014.

Characteristic	n* = 1073	Mean	SD**
Maternal Age (years)	1073	24.18	5.13
Gestational age at initial ANC booking (weeks)	716	22.78	7.19
Infant gestation at birth (weeks)	946	37.16	2.92
Birth weight (grams)	1069	2675.48	616.16
Infant length (cm)	948	48.86	4.06
Infant head circumference (cm)	958	33.21	2.21

* sample size

** Standard Deviation

358 observations had information on gestation age at first ANC

128 observations had missing information on infant gestational age at birth

5 observations had missing information on birth weight

126 observations had missing information on infant length

116 observations had missing information on infant head circumference

We analysed continuous variables to determine the relationship between low birth weight (LBW) and normal birth weight (NBW). LBW babies had a mean gestation age of 35.60 weeks, compared to mean gestational age of 37.90 weeks for NBW. The infant length and head circumference of LBW babies was less than that of NBW infants (Table 2).

**Table 2:** Comparison of means for continuous variables for 1073 mothers who delivered in four Tshwane hospitals, Gauteng, South Africa in 2014.

Characteristic	n*	Mean	SD**	P value
**Maternal Age (years)**	**1069**			
NBW^a^	552	24.06	5.10	0.483
LBW^b^	517	24.22	5.11	
**Gestation at first ANC (weeks)**	**713**			
NBW	376	22.97	7.13	0.447
LBW	337	22.56	7.24	
**Gestation age (weeks)**	**954**			
NBW	524	37.90	4.03	<0.001
LBW	430	35.60	3.75	
**Infant length (cm)**	**944**			
NBW	510	50.67	3.64	<0.001
LBW	434	46.68	3.80	
**Infant head circumference (cm)**	**954**			
NBW	514	33.26	1.34	<0.001
LBW	440	32.01	2.34	

* Sample size

** Standard deviation

^a^ Normal birth weight

^b^ Low birth weight.

4 observations had missing information on maternal age

360 observations had information on gestation age at first ANC

119 observations had missing information on infant gestational age at birth

129 observations had missing information on infant length

119 observations had missing information on infant head circumference

Sociodemographic factors associated with LBW were maternal age, race of the mother and the residence of the mother. Women older than 20 years (n = 412, 38.54% of all LBW deliveries) were at risk of LBW delivery (Odds ratio [OR] 1.33, CI 1.00 to 1.77). Black African women had an OR 2.67 (CI 1.23 to 5.81) of delivering a LBW infant, compared to white mothers, while the risk of LBW for Asian women was OR 5.55, CI 1.77 to 17.37 compared to white mothers. Marital status was not associated with LBW, and the place where the women delivered was not associated with LBW (Table 3).

**Table 3:** Crude odds ratios for low birthweight by socio-demographic characteristics for 1073 mothers delivering in four Tshwane hospitals, Gauteng, South Africa in 2014.

Characteristic	Total births 1073		Low birth weight births		Crude Odds Ratio (95 % CI*)
	n**	%	n	%	
**Maternal age**					
<20 years	245	22.92	105	9.82	Reference
> 20–35 years	824	77.08	412	38.54	**1.33 (1.00 to 1.77)**
**Marital status**					
Single	312	77.61	131	32.75	0.84 (0.69 to 2.01)
Married	67	16.67	31	7.75	reference
Stable relationship	23	5.76	10	2.25	1.14 (0.48 to 2.73)
**Race**					
Black African	693	92.28	340	45.27	**2.67 (1.23 to 5.81)**
Asian	24	3.20	16	2.13	**5.55 (1.77 to 17.37)**
White	34	4.53	9	1.20	Reference
**Residence**					
Rural	668	63.20	329	31.13	1.05 (0.81 to 1.36)
Semi–urban	34	3.22	9	0.85	**0.39 (0.17 to 0.86**)
Urban	355	33.59	170	16.08	Reference
**Facility**					
Mamelodi Hospital	300	28.06	146	13.66	Reference
Pretoria West Hospital	182	17.03	72	6.74	0.69 (0.47 to 1.00)
Laudium CHC	259	24.23	139	13.00	1.22 (0.87 to 1.70)
Kgabo CHC	328	30.68	160	14.97	1.00 (0.73 to 1.37)

* Confidence interval

** sample size

4 observations had missing information on maternal age

671 observations had missing information on marital status

332 observations had missing information on race

16 observations had missing information on residence

4 observations had missing information on facility

Prenatal factors associated with LBW were ANC attendance, the number of ANC visits, not having a syphillis test and a positive HIV status. Almost five percent (4.80%) of mothers who did not attend ANC delivered a LBW infant. Mothers who did not attend ANC had increased risk (OR 2.65, CI 1.58 to 4.43) of delivering a LBW baby compared to those who attended ANC. Women who did not attend ANC, or had missing information on the ANC visits had increased risk of delivering a LBW infant (OR 3.76,CI 2.27 to 6.22) compared to women who attended more than 5 ANC visits. Women who attended ANC 1 to 4 times, had an increased risk (OR 1.72 CI 1.22 to 2.43) of delivering LBW babies compared to women who attended more than 5 times. Eight percent of women tested positive (using RPR, rapid plasma reagin test) or had unknown syphillis status. Women who tested positive for syphyilis or had unkown syphilis status had a greater risk of delivering a LBW baby (OR 2.27, CI 1. 54 to 3. 34) compared to women who tested negative for syphillis. About 11.36% of women tested positive for HIV, and had an increased risk of delivering a LBW infant (OR 1.44 CI 1.07 to 1.94) compared to women who tested negative for HIV. While women who had unknown HIV status had an increased risk of delivering a LBW infant, (OR 2.00 CI 0.90 to 4.43), the association was not statistically significant, see Table 4.

**Table 4:** Crude odds ratios for low birthweight by prenatal Factors for 1073 mothers who delivered in four Tshwane hospitals, Gauteng, South Africa, 2014.

Characteristic	Total births 1073			Low birth weight births		Crude Odds ratio (95 % CI*)
	n**		%	n	%	
**Attended ANC**^a^						
Yes	994		93.16	462	43.46	Reference
No	73		6.84	41	4.80	**2.65 (1.58 to 4.43)**
**Number of ANC visits**						
0	113		10.57	76	7.11	**3.76 (2.27 to 6.22)**
1–4	786		73.53	381	35.64	**1.72 (1.22 to 2.43)**
>5	170		15.90	60	5.61	Reference
**Parity**						
0	267		25.07	118	11.08	0.77 (0.58 to 1.03)
1–2	656		61.60	331	31.08	Reference
>2	142		13.33	66	6.20	0.85 (0.59 to 1.22)
**Previous LBW**^b^						
Yes	15		6.61	10	4.41	2.41 (0.79 to 7.31)
No	212		93.39	96	42.29	Reference
**Gestation at 1**^**st**^ **ANC visit**						
≤20 weeks	288		40.39	141	19.78	Reference
>20 weeks	425		59.61	196	27.49	1.12 (0.8 to 1.51)
**Syphilis tested**						
Yes	928		87.96	421	39.91	Reference
No	127		12.04	83	7.87	**2.27 (1.54 to 3.34)**
**HIV status**						
Negative	818		76.81	375	35.21	Reference
Positive	220		20.66	121	11.36	**1.44 (1.07 to 1.94)**
Unknown	27		2.54	17	1.60	2.00 (0.90 to 4.43)

* Confidence intervals

** sample size

^a^ antenatal care

^b^ low birth weight

6 observations had missing information on ANC attendance

4 observations had missing information on number of ANC visits

8 observations had missing information on parity

846 observations had missing information on history of previous LBW

18 observations had missing information on being tested for syphilis

8 observations had missing information on HIV status

Obstetric risk factors associated with LBW included women with preeclampsia (OR 3.74, CI 1.04 to 8.84) and premature rupture of membranes (PROM) (OR 6.74, CI 2.27 to 20.02). Anaemia, hypertension and infections were not significant predictors of LBW deliveries. Most obstetric risk factors had missing values, and were excluded from the analysis.

Low birth weight babies had a greater risk of mortality (OR 5.89 CI 1.70 to 20.34) than NBW babies. For infants with congenital anomalies, the risk of LBW was OR 3.30 (CI 1.19 to 9.17). Babies who were born premature had a higher risk of LBW (OR 7.31, CI 5.33 to 10.04) than babies born at term. Male infants were less likely to be LBW (OR 0.77, CI 0.60 to 0.98) (Table 5).

**Table 5:** Crude odds ratios for low birth weight by obstetrical complications of current pregnancy for 1073 mothers who delivered in four Tshwane hospitals, Gauteng, South Africa in 2014.

Characteristic	Total births		Low birth weight births		Crude Odds ratio
	n*	%	n	%	(95 % CI**)
**Anaemia**					
Yes	28	7.22	16	4.12	1.88 (0.86 to 4.10)
No	360	92.78	149	38.40	Reference
**Hypertension**					
Yes	28	7.22	16	4.12	1.88 (0.86 to 4.10)
No	360	92.78	149	38.40	Reference
**Preeclampsia**					
Yes	16	1.96	11	1.35	**3.04 (1.04 to 8.84)**
No	801	98.04	336	41.13	Reference
**PROM**^a^					
Yes	23	2.82	19	2.33	**6.74 (2.27 to 20.02)**
No	794	97.18	328	40.15	Reference
**Infections**^b^					
Yes	47	5.72	21	2.56	1.08 (0.60 to 1.96)
No	774	94.28	330	40.19	Reference
**Delivery type**^c^					
Spontaneous vertex	1,011	94.66	487	45.60	Reference
Elective C/S^d^	16	1.50	6	0.56	0.64 (0.23 to 1.78)
Emergency C/S	41	3.84	24	2.25	1.51 (0.80 to 2.86)
**Birth status**					
Alive	1,044	98.21	496	46.66	Reference
Dead	19	1.79	16	1.51	**5.89 (1.70 to 20.34)**
**Congenital anomalies**					
Yes	20	1.89	15	1.41	**3.30 (1.19 to 9.17)**
No	1,041	98.11	495	46.65	Reference
**Infant sex**					
Female	527	49.76	271	25.59	Reference
Male	532	50.24	239	22.57	**0.77 (0.60 to 0.98)**
**Infant maturity status**					
Preterm	295	31.22	201	21.27	**7.31 (5.33 to 10.04)**
Term	650	68.78	226	23.92	Reference

* Sample size

** Confidence intervals

^a^ Premature rupture of membranes (PROM)

^b^ Infection = sexually transmitted (HIV excluded) and urinary tract infection as documented by the nurses and doctors in the files

^c^ This study group did not have breech and instrumental delivery

^d^ Caesarean Section

684 observations had missing information on Anaemia

684 observations had missing information on hypertension

255 observations had missing information on preeclampsia

255 observations had missing information on PROM

251 observations had missing information on delivery type

7 observations had missing information on birth status

9 observations had missing information on congenital disorders

11 observations had missing information on infant sex

127 observations had missing information on infant maturity

In adjusted analysis, maternal age was associated with LBW (adjusted odds ratio [AOR] 12.20, CI 3.90 to 38.02) for older women compared to younger women. Women who had fewer than 5 ANC visits also remained significantly associated with LBW (AOR 1.30, CI 1.06 to 1.61) compared to those who attended 5 or more visits. Additionally, PROM remained significantly associated with LBW (OR 7.33, CI 2.43 to 22.12). Preterm delivery also remained associated with LBW (OR 7.15, CI 5.18 to 9.89) and male infants remained less likely to be LBW (OR 0.52, CI 0.52 to 0.92). See Table 6.

**Table 6:** Adjusted* odds ratios for low birth weight risk factors for mothers who delivered in Tshwane District, Gauteng, South Africa, in 2014.

Low birth weight	Adjusted* Odds Ratio	(95 % CI**)
Maternal age	12.20	**3.90 to 38.02**
Race	0.84	0.41 to 1.71
Residence	0.94	0.50 to 1.73
ANC^a^ attendance	1.29	0.10 to 16.65
Number of ANC visits	1.30	**1.06 to 1.61**
Syphilis tested	1.27	0.69 to 2.35
HIV status	1.61	**1.13 to 2.29**
Preeclampsia	2.09	0.48 to 9.37
PROM^b^	7.33	**2.43 to 22.12**
Birth status	3.45	0.73 to 16.31
Congenital anomalies	2.54	0.80 to 8.04
Infant sex	0.70	**0.52 to 0.92**
Infant maturity status	7.15	**5.18 to 9.89**

* Adjusted for other sociodemographic, prenatal and obstetric causes

** Confidence intervals

^a^ Antenatal care

^b^ Premature rupture of membranes

## Discussion

We conducted a case control study to identify the factors associated with giving birth to a LBW baby in Tshwane, South Africa. In our sample of 1073 mothers, 77% were between the 20 and 35 years old. Young mothers (<20 years) did not have an increased risk of delivering a LBW baby. In this study, LBW was associated with older maternal age; preterm birth; inadequate prenatal care; maternal HIV and syphilis infections; premature rupture of membranes and preeclampsia; and infant sex.

In our study, older maternal age was a risk factor for LBW. In contrast to our study, some studies have reported that teenage mothers are more likely to deliver a LBW infant compared to adult mothers [20, 21]. Ngoma et al and Althabe report that younger teenage mothers (< 16 years) are at increased risk of LBW compared to older teenage mothers [6, 7]. Hoque and Hoque (2010) found that there was no difference in adverse outcomes (LBW) related to maternal age in rural areas of South Africa [17]. Similarly, another study by Hoque et al (2014) noted that maternal age did not influence LBW delivery in a large tertiary hospital in Tshwane [16]. Our study reports the opposite findings, that older mothers have increased risk of delivering a LBW infant compared to teenage mothers. Our study differed from Hoque et al. (2014) because we sampled for a 1:1 ratio of cases to controls while they described the birth outcomes in the whole registry for a specific time. To identify the effect of maternal age on LBW, we need carefully designed experiments that will avoid bias. As expected, preterm birth is the main risk factor for LBW in our study, in agreement with other studies [7, 8, 22]. While preterm birth is the main predictor of LBW, the factors that predispose mothers to preterm birth differ on a case by case basis. Preterm birth has been linked to medications taken during pregnancy and infections of the mother during pregnancy [12]. Preterm delivery has also been associated with very young maternal age [7], and when combined with very LBW, is a significant predictor of neonatal mortality and morbidity [16]. Adverse birth outcomes, LBW and pre-term delivery have also been associated with not attending antenatal (ANC) care [23].

In our study, women who attended 5 or more ANC visits had a reduced risk of giving birth to a LBW baby. Antenatal care needs to be encouraged, especially in poor socio-economic settings [13, 24]. In Uganda, Bayo *et al* [25] did not find any significant association between LBW outcomes and ANC attendance in teenage mothers in a single site study. Other multi-site studies from Uganda by Kumar *et al* [26] and Mcdiehl *et al* [24] found that mothers who did not attend ANC had a higher risk of adverse birth outcomes. During ANC visits, potential risk factors are screened for, and preventive interventions to avoid LBW and other poor birth outcomes are often implemented. These risk factors may remain undiagnosed if women do not attend ANC visits during pregnancy. Similarly, fewer ANC visits may mean that medical and obstetrical risk factors, as well as infections, are missed or not managed adequately during pregnancy [10, 14]. Of concern in our study is the low proportion (~20%) of women who attended 5 or more ANC visits. The importance of ANC visits need to be promoted in this population and factors that limit the attendance of ANC need to be assessed.

In our study, we identified HIV as a possible risk factor for LBW, similar to some studies [16, 10]. South Africa has a high HIV incidence [27], which has fallen in response to interventions such as HIV awareness programs and the free antiretroviral therapy (ART) [19]. These preventive strategies may explain the low frequency (~ 21%) of HIV in our study. According to the South African Demographic Health Survey 2016, 93% of South Africans were aware of HIV/AIDS and testing programs. Therefore, HIV negative women are more likely to adopt measures that reduce the risk of contracting HIV by engaging in safer sex [28]. Women who have HIV may be more prone to delivering [16] LBW babies due to the effects of antiretroviral therapies (ART) [14]. Besides, HIV is an immune altering condition; patients are prone to different diseases as well as undernutrition, which is a known risk factor for LBW [6, 10]. Thus, malnutrition may predispose HIV infected women to giving birth to LBW babies [29].

While our adjusted analysis did not find an association between syphilis infection and LBW, our crude analysis highlighted a potential influence, similar to previous studies [30]. In this population, infections continue to increase the risk for LBW. Attending ANC care is vitally important because South African women are screened for syphilis during ANC. If a mother tests positive for syphilis, she is treated with Benzanthine Penicillin, to prevent congenital syphilis, and other poor birth outcomes [31].

Premature rupture of membranes is associated with both preterm delivery [10, 13, 32], which is associated with LBW [11]. If PROM occurs, the loss of amniotic fluid restricts foetal growth and results in the birth of a prematurely born LBW infant [11]. In our study, we also noticed that preeclampsia was associated with LBW according to crude analysis, similar to previous studies [32]. Hypertension was not associated with LBW in our study due to small sample size, but has been reported to be significantly associated with LBW in other studies [13].

We found that male babies were less likely to be born with LBW, which is consistent with some studies [33–34]. However, a conflicting finding was reported in Zimbabwe where female babies were less likely to be born with LBW [16]. Therefore, there is need for more studies in Tshwane to further investigate this finding.

Our study was limited by the use of self-reported LMP to determine gestational age. Ultrasound scans would estimate gestational age more accurately [35], but their use is limited in low middle income and developing countries such as South Africa. As with many retrospective studies, missing data might influence the results negatively. Though our sample size was fairly adequate (1073 mothers), most of the obstetric and sociodemographic data were missing. Ideally, we would like to conduct a prospective study to reduce missing data. In addition, the generalisability of our findings could be improved by including data from more provinces in South Africa. Despite these limitations, our study has several strengths. The study had a reasonable sample size and was conducted in the largest district in South Africa which has a diverse population. We sample data from several hospitals of different levels within the Tshwane District, Gauteng Province, South Africa, providing a good representation of mothers in the district.

## Conclusion

Low birth weight continues to affect the health and wellbeing of infants. By recognising the risk factors that contribute to LBW, interventions can be tailored to address those factors. Low birth weight is a public health concern of unknown magnitude in Africa, and particularly in Tshwane District, South Africa [36]. Therefore, it is important to create an awareness of LBW amongst mothers so that they can take control of their lives and that of their unborn child during pregnancy. Additionally, ANC attendance should be advocated through different platforms such as community oriented primary care and social media (mom—connect) for prompt interventions to prevent LBW. Lowering the incidence of LBW will help in reducing infant morbidity and mortality in South Africa, and similar settings.
